# Family Functioning and Adolescent Mental Health: The Mediating Role of Bullying Victimization and Resilience

**DOI:** 10.3390/bs14080664

**Published:** 2024-08-01

**Authors:** Juan Zhang, Xiang Duan, Yiwen Yan, Yuxin Tan, Taimin Wu, Yaofei Xie, Bing Xiang Yang, Dan Luo, Lianzhong Liu

**Affiliations:** 1 Affiliated Wuhan Mental Health Center, Tongji Medical College of Huazhong University of Science and Technology, Wuhan 430012, China; zhangjuanwhjw@163.com (J.Z.); tymon555@163.com (T.W.); xieyf65@foxmail.com (Y.X.); 2 Department of Psychology, Institute of Education, China University of Geosciences, Wuhan 430074, China; yanyiwenlucky@163.com; 3 Student Mental Health Education Center, China University of Geosciences, Wuhan 430074, China; hbu_tyx2012@163.com; 4 Center for Wise Information Technology of Mental Health Nursing Research, School of Nursing, Wuhan University, Wuhan 430071, China; 00009312@whu.edu.cn (B.X.Y.); luodan@whu.edu.cn (D.L.); 5 Wuhan Wudong Hospital (Wuhan Second Mental Hospital), Wuhan 430084, China

**Keywords:** family functioning, bullying victimization, resilience, adolescent mental health, gender differences

## Abstract

This study aimed to understand the current state of adolescent mental health, explore the mediating effect of bullying victimization and resilience in the relationship between adolescent family functioning and mental health, and investigate gender differences in this association. A total of 4319 students (2347 boys and 1972 girls) completed the questionnaire. Mediating effects were analyzed using the framework of structural equation modeling and bootstrapping. The results revealed that family functioning is significantly associated with adolescent mental health, and that bullying victimization and resilience have significant independent and chain mediating effects on this relationship. Multiple group analysis revealed that the independent mediating role of resilience was more significant for male adolescents. Furthermore, the chain-mediated effects of bullying victimization and resilience were observed only in the relationship between family functioning and mental health in male adolescents. To improve the mental health of adolescents, special attention should be given to the impact of family life on adolescents’ school life. Early detection and intervention for adolescents with poor family functioning are also important to effectively prevent bullying victimization and reduce the emergence of mental health problems.

## 1. Introduction

Adolescence is a critical stage in life for an individual’s growth, learning, and future development. However, mental health issues among adolescents are increasingly significant in modern society [1]. The World Health Organization reported that approximately 20% of children and adolescents worldwide experience mental health issues, such as anxiety, depression, self-harm, stress, and behavioral problems [2]. High school is a period of rapid physical and psychological changes for adolescents. Studies have shown that academic and relationship challenges during this time can lead to greater mental health problems [3]. Therefore, the mental health of adolescents, particularly high school students, has become a significant social concern.

Depression and anxiety are frequently occurring symptoms of mental health issues in adolescents and are crucial indicators for evaluating adolescent mental health [4]. These symptoms can have significant consequences for the quality of life and development of adolescents, particularly in the areas of academics, family relationships, socialization, and physical health. When adolescents experience depression and anxiety, they often exhibit poor concentration and decreased motivation to learn [5], and may feel lonely and socially avoidant. In the long term, these mental health issues may also impact adolescents’ immune system functioning and increase the risk of developing chronic diseases [6].

The bioecological model of human development proposes that an individual’s development depends on interactions between the individual (individual system), family, school, community, and other factors (e.g., macrosystems, temporal systems) [7]. Moreover, it has been proposed that the relationships and socialization of adolescents within the family can significantly impact their relationships with others in the school environment and their personal development [8]. Accordingly, this study aimed to examine the interrelationships between family, school, and individual factors in order to gain insight into how these factors influence adolescent mental health and to develop a more effective approach to promoting adolescent mental health.

### 1.1. The Influence of Family Functioning on Mental Health

As the primary environment in which a child grows up, the family plays a crucial role in an adolescent’s physical and mental development. According to multilevel family systems theory, the family is considered an interdependent system in which interactions and changes among family members not only affect the equilibrium of the entire family system but also have a profound impact on the psychology and behavior of each member [9]. Some studies have demonstrated that the quality of family functioning has a direct impact on their mental health [10]. Adolescents’ sense of independence, self-esteem, security, and self-confidence are significantly influenced by their families. Additionally, intimate relationships and conflicts within the family functioning can affect children’s social skills and ability to cope with mental stress. Tamplin, Goodyer, and Herbert revealed that families of depressed individuals face challenges in multiple areas [11]. Poor family functioning has been linked to depression and anxiety in children [12]. Poorly functioning families increased the recurrence rates and shortened the recurrence intervals of depression in adolescents [13]. Additionally, adaptability and cohesion in family functioning have been associated with anxiety in children [14].

### 1.2. The Mediating Role of Bullying Victimization

Bullying is a form of peer violence that involves repeated and intentional physical or emotional aggression aimed at controlling or harming another person [15]. It can have significant negative impacts on adolescents. Bullying victimization has become a significant social issue. However, intervening directly in school bullying among adolescents can be challenging, and victims may be hesitant to report or seek help due to fear of further bullying or social ostracism. Therefore, identifying other modifiable factors may be a more reasonable approach to preventing bullying victimization among adolescents and promoting mental health.

Research has demonstrated that children who feel understood and supported at home are more likely to display positive social behaviors at school, which reduces the risk of bullying [16]. According to a review study, poor family functioning may increase a child’s risk of being bullied [17]. Studies have shown that high school students who are victims of school bullying score lower on family support. Additionally, there may be child rejection and abuse in the home [18]. Researchers suggest that the family climate contributes to bullying victimization by influencing students’ social relationships with teachers and peers in school [19]. Källmén and Hallgren found that boys who experienced bullying at school reported a fourfold increase in the prevalence of mental disorders compared to those who did not experience bullying [20]. Similarly, girls who were bullied reported a 2.5 times greater prevalence of mental disorders. Research has shown that lower family support is associated with more severe depression in girls than in boys [21]. Additionally, bullying victimization has a greater impact on depression in female students than in male students [22]. Therefore, it is hypothesized that bullying victimization mediates the relationship between family functioning and resilience, and that there are gender differences.

### 1.3. The Mediating Role of Resilience

Resilience is one of the hottest topics in the field of positive psychology [23]. Resilience refers to an individual’s capacity to adapt, recover, and grow in response to life’s stresses, challenges, changes, or traumas [24]. It enables a person to cope with adverse experiences and reduces the likelihood of mental health problems [25]. According to a Korean study, there is a negative correlation between depression and family functioning, particularly family cohesion. Resilience was found to act as a mediator, partially explaining the relationship between family functioning and depression [26]. Research has demonstrated that resilience plays a mediating role in the association between bullying victimization and anxiety. Specifically, emotional regulation, family support, and interpersonal assistance have been identified as significant mediators [27]. A significant negative correlation was found between resilience and depression and anxiety. Higher levels of resilience were associated with less severe symptoms of depression and anxiety. Resilience was also found to partially mitigate the effects of bullying on depression and anxiety [28]. Lin et al. found that resilience is protective for adolescents who have experienced bullying, reducing the risk of depression [29]. Empirical evidence suggests that positive family functioning contributes to building resilience in adolescents and improving their mental health.

### 1.4. The Current Study

A substantial body of studies have demonstrated that adolescent mental health problems are associated with family functioning, bullying victimization, and resilience [30,31,32]. However, the mechanisms by which these variables collectively influence each other are not yet clear. This study aimed to examine the impact from three levels (family, school, and individuals) on adolescent mental health and to determine whether gender differences exist. We utilized structural equation modeling to simultaneously incorporate multiple variables and developed three research hypotheses: (a) Adolescent bullying victimization and resilience independently mediate the association between family functioning and adolescent mental health; (b) Bullying victimization and resilience act as a chain mediator between family functioning and adolescent mental health; (c) There were gender differences in the independent and chained mediating effects of bullying victimization and resilience in the relationship between family functioning and mental health, which were more significant among girls. The goal was to better understand adolescent mental health and identify important risk factors, such as bullying victimization, for intervention.

## 2. Methods

### 2.1. Data and Sample

This study was conducted as part of the Students’ Mental Health Network (SMHN), a project implemented in a major city in central China in January 2023. A convenience sampling method was used to select 5345 students from four high schools in a first-tier city in central China for a questionnaire survey. The investigators were uniformly trained and organized the students to complete the survey on-site as a class with the cooperation of the schools. Prior to the survey, the students received an explanation of the study’s purpose and nature, as well as instructions on how to complete the form. Participants accessed the SMHN’s proprietary platform to complete the anonymous self-report questionnaires and submitted them on the site. During this process, the teachers were asked to recuse themselves. The Ethics Committee of the Affiliated Wuhan Mental Health Center approved the study. Ultimately, 4319 students completed the survey with no missing information, resulting in a valid recall rate of 80.8%. All of these students were included in the analysis.

### 2.2. Measurements

#### 2.2.1. Sociodemographic Information

The researcher designed the questionnaire to collect sociodemographic information, including gender, grade level, whether the child was an only child, and the family’s socio-economic status.

#### 2.2.2. Family Functioning

The Family APGAR Scale was utilized to evaluate the family functioning of the adolescents, which was initially proposed by Smilkstein in 1978 [33]. This scale comprises five questions that assess family adjustment, partnership, growth, emotion, and intimacy. The scale’s statements pertain to emotional expression, communication, and interaction between the respondent and family members. A 3-point Likert scale (ranging from 0 (almost never) to 2 (almost always)) was used to measure satisfaction with family functioning. The score reflects the level of satisfaction with family functioning, with higher scores indicating greater satisfaction. The Chinese version of the scale has high reliability and validity, with a Cronbach’s α of 0.843 and a 2-week retest reliability of 0.724 [34]. In this study, the Cronbach’s α was 0.906.

#### 2.2.3. Bullying Victimization

This research utilized the Chinese version of the Olweus Child Bullying Questionnaire, revised by Zhang, Wu, and Jones in 1999, to assess students’ bullying victimization [35]. Specifically, we selected the subscale that measures the type of bullying, which consists of six items. Bullying was rated on a five-point scale based on the frequency of occurrence, ranging from 0 (none this semester) to 4 (several times a week). According to the previous classification criteria, students were considered victims of bullying if they scored ≥ 2 on any one of the six bullying topics, indicating a frequency of occurrence of more than 2–3 times per month. The Cronbach’s α for this study was 0.838.

#### 2.2.4. Resilience

This study measured the resilience of adolescents using the 10-item Connor–Davidson Resilience Scale (CD-RISC-10). The scale was simplified from the 25-item CD-RISC by Campbell-Sills et al. in 2007 [36]. It consists of 10 items and is scored on a 5-point Likert scale, ranging from 1 for “never” to 5 for “always” [37]. The resilience scale’s total score is the sum of each item’s score. A higher total score indicates greater resilience. The Chinese version of the CD-RISC-10 used in this study had high reliability and validity, with a Cronbach’s α of 0.951.

#### 2.2.5. Depression

The Patient Health Questionnaire-9 (PHQ-9) was utilized to assess depression in adolescents, which was developed by Kroenke and colleagues in 2001 [38]. The nine items align with the DSM criteria for major depression: (1) anhedonia; (2) depressed mood; (3) insomnia or hypersomnia; (4) fatigue; (5) changes in appetite; (6) feelings of guilt, self-blame, or worthlessness; (7) difficulty concentrating; (8) psychomotor agitation or retardation; and (9) suicidal ideation or self-harm. Each item is rated on a 4-point scale ranging from 0 to 3 (0 = never; 1 = several days; 2 = more than half the time; 3 = almost every day). The total score ranges from 0 to 27. A score of 10 or higher indicates the presence of more than moderate depressive symptoms, which is the most commonly used cutoff point for major depression [39]. The Chinese version of the PHQ-9 used in this study demonstrated high reliability and validity, with a Cronbach’s α of 0.899.

#### 2.2.6. Anxiety

The Generalized Anxiety Disorder Scale-7 (GAD-7) is a self-report questionnaire developed by Spitzer and colleagues in 2006 for the rapid detection of anxiety disorders [40]. Participants answer seven questions about anxiety-related problems experienced in the past two weeks. Total scores range from 0 to 21. The GAD-7 has been found to have the best sensitivity and specificity for detecting GAD compared to a structured psychiatric interview when using a 10-point threshold. The Chinese version of the GAD-7 used in this study demonstrated high reliability and validity, with a Cronbach’s α of 0.931.

### 2.3. Data Analysis

Descriptive statistics and correlation analysis of the data were conducted using SPSS 24.0 software. Continuous variables are expressed as the mean ± standard deviation, and categorical variables are expressed as numbers and percentages. Mental health was compared across demographic variables using the chi-square test or ANOVA. The relationships between family functioning, bullying victimization, resilience, depression, and anxiety were reported using Pearson’s correlation. This study utilized Mplus 8.3 software to conduct structural equation modeling (SEM) to examine the potential mediating role of bullying victimization and resilience in the relationship between family functioning and mental health. The control variables included grade, gender, and economic class of the family. Multigroup analyses were conducted to test for potential gender differences in the model. The model fit was evaluated using commonly used fit indices. The data model was considered to have a good fit when both the comparative fit index (CFI) and the Tucker–Lewis index (TLI) were greater than or equal to 0.90, and the root mean square error of approximation (RMSEA) was less than or equal to 0.08 [41]. As the results of the chi-square test are strongly correlated with the sample size, they are generally considered less reliable, and therefore, other indicators are more valuable in assessing model fit [42]. The regression equation parameters are indicated by single arrows. The validation factor analysis, model fit test, and path analysis were completed using the maximum likelihood method. Bootstrap analysis was performed to test the mediating effect at the test level of α = 0.05, with *p* < 0.05 indicating a statistically significant difference.

## 3. Results

### 3.1. Common Method Bias

To ensure objectivity, a common method bias test was conducted because the data were obtained from subjects’ self-reports. Harman’s one-way test was used, resulting in five factors with eigenvalues greater than 1, explaining a total of 65.85% of the variance. The first factor, which explained 35.11% of the variance, did not exceed the critical value of 50% [43]. Therefore, it can be concluded that there was no significant common method bias in this study.

### 3.2. Descriptive Statistics

The total number of participants was 4319, including 2347 (54.3%) male adolescents and 1972 (45.7%) female adolescents, 2732 (63.3%) only children and 1587 (36.7%) non-only children, and 271 (6.3%) victims of bullying (Table 1). The mean PHQ-9 score for adolescents was 4.97, with 649 having depressive symptoms, a detection rate of 15.0%; the mean GAD-7 score was 4.72, with 561 having anxiety symptoms, a detection rate of 13.0%. There were significant differences in the depression and anxiety scores among adolescents of different genders, grades, and family economies (*p* < 0.01), with girls scoring higher on depression and anxiety than boys. There was no significant difference in scores on the depression and anxiety scales between whether the child was an only child or not (*p* > 0.05) (Table 1).

### 3.3. Correlation Analyses

Table 2 shows that family functioning was negatively correlated with bullying victimization (*r* = −0.100, *p* < 0.01), depression (*r* = −0.431, *p* < 0.01), and anxiety (*r* = −0.382, *p* < 0.01), and positively correlated with resilience (*r* = 0.394, *p* < 0. 01). Bullying victimization was positively correlated with depression (r = 0.184, *p* < 0.01), anxiety (*r* = 0.171, *p* < 0.01) and negatively correlated with resilience (*r* = −0.106, *p* < 0.01); resilience was negatively correlated with depression (*r* = −0.459, *p* < 0.01) and anxiety (*r* = −0.439, *p* < 0.01); depression and anxiety were significantly positively correlated (*r* = 0.794, *p* < 0.01).

### 3.4. Mediation Analyses

#### 3.4.1. Family Functioning and Depression

**Figure 1:**
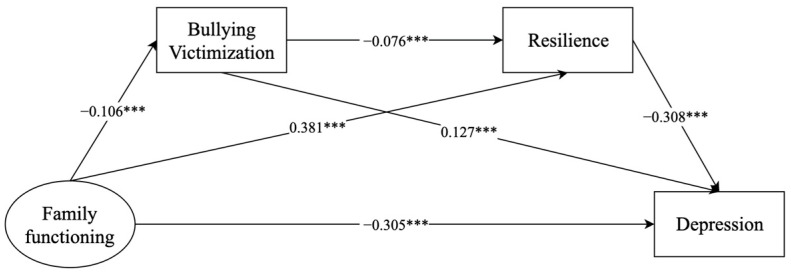
Mediating effects of bullying victimization and resilience on the relationship between family functioning and depression. *** *p* < 0.001.

Figure 1 displays the model constructed with family functioning as an exogenous variable and bullying victimization, resilience, and depression as endogenous variables. The model fit indices were met, *χ*^2^ = 334.219 (*df* = 31, *p* < 0.001), CFI = 0.982, TLI = 0.969, SRMR = 0.058, and RMSEA = 0.048 (90% CI = 0.043, 0.052). The results showed that family functioning was positively associated with adolescent depression (*β* = −0.305, 95% CI: −0.338 to −0.272) and that bullying victimization and resilience independently mediated the relationship between family functioning and adolescent depression (bullying victimization: *β* = −0.013, 95% CI: −0.020 to −0.009; resilience: *β* = −0.118, 95% CI: −0.134 to −0.102). In addition, the chain mediation of bullying victimization and resilience also played a role in the relationship between family functioning and adolescent depression (*β* = −0.002, 95% CI: −0.003 to −0.001) (Table 3).

#### 3.4.2. Family Functioning and Anxiety

**Figure 2:**
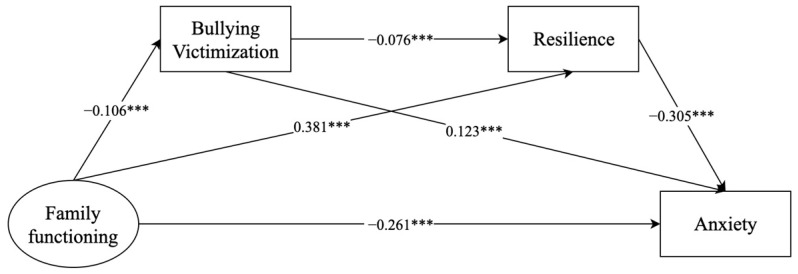
Mediating effects of bullying victimization and resilience on the relationship between family functioning and anxiety. *** *p* < 0.001.

Figure 2 presents a model that illustrates the relationship between family functioning and anxiety, which is mediated by bullying victimization and resilience. The model fit metrics were all met (*χ*^2^ = 335.148 (*df* = 31, *p* < 0.001), CFI = 0.981, TLI = 0.969, SRMR = 0.058, and RMSEA = 0.048 (90% CI = 0.043, 0.052)). The results revealed a significant association between family functioning and adolescent anxiety (*β* = −0.261, 95% CI: −0.297 to −0.225), and this association was found to be mediated by bullying victimization or resilience (bullying victimization: *β* = −0.013, 95% CI: −0.019 to −0.008; resilience: *β* = −0.116, 95% CI: −0.133 to −0.101). Furthermore, family functioning was found to be linked to adolescent anxiety through the chain mediation of bullying victimization and resilience (*β* = −0.002, 95% CI: −0.004 to −0.001) (Table 4).

#### 3.4.3. Gender Differences

**Figure 3:**
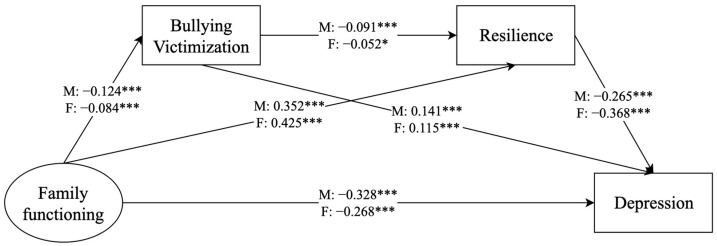
Analysis of the mediating role of bullying victimization and resilience on the relationship between family functioning and depression, with a focus on gender. * *p* < 0.05, *** *p* < 0.001. male (M), female (F).

**Figure 4:**
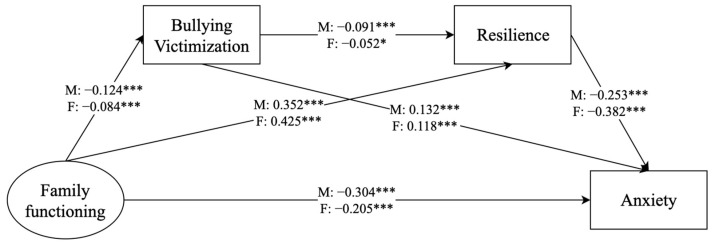
Analysis of the mediating role of bullying victimization and resilience on the relationship between family functioning and anxiety, with a focus on gender. * *p* < 0.05, *** *p* < 0.001. male (M), female (F).

To examine potential gender differences, we divided the sample into male and female groups for analysis (Figure 3 and Figure 4). The model fit indicators were met for depression (*χ*^2^ = 382.613 (*df* = 60, *p* < 0.001), CFI = 0.980, TLI = 0.971, SRMR = 0.063, RMSEA = 0.050, 90% CI = 0.045, 0.055) and anxiety (*χ*^2^ = 380.481 (*df* = 60, *p* < 0.001), CFI = 0.980, TLI = 0.971, SRMR = 0.063, RMSEA = 0.050, 90% CI = 0.045, 0.055). The results revealed that the chain mediation of bullying victimization and resilience played a significant role in the correlation between family functioning and depression/anxiety in male adolescents but not in female adolescents. Furthermore, the remaining indirect effects were significant in both groups (Table 5 and Table 6).

Multiple group analysis was used to explore whether the mediating effects of the modeled pathway differed between boys and girls. The test results indicated no gender differences in the pathway of “family functioning–bullying victimization–depression/anxiety” (depression: Wald test *χ*^2^ = 2.241, *p* > 0.05; anxiety: Wald test *χ*^2^ = 1.745, *p* > 0.05). However, significant gender differences were found in the pathway of “family functioning–resilience–depression/anxiety” (depression: Wald test *χ*^2^ = 14.000, *p* < 0.001; anxiety: Wald test *χ*^2^ = 17.285, *p* < 0.001). 

## 4. Discussion

Ecological Systems Theory highlights that family, school, and individual systems are not isolated entities; rather, their interactions collectively impact adolescents’ mental health. In this study, we use structural equation modeling to examine how these systems interact to influence adolescents’ psychological well-being. Previous research findings indicated that good family functioning has a positive association with adolescent mental health. Bullying victimization and resilience not only mediated this relationship independently, but also had a chain mediating role in the relationship. In the analysis of multiple groups of comparisons, it was found that the mediating role of resilience in the relationship between family functioning and mental health was more significant in male adolescents. Furthermore, after the sample was divided into male and female groups, the chain mediation of bullying victimization and resilience was found to exist between family functioning and mental health only in the male group.

### 4.1. Depression and Anxiety in Adolescents

This survey revealed that the detection rates of depression and anxiety among high school students in urban central China were generally consistent with the findings of [44], but in a study of depression and anxiety among adolescents in Beijing, the detection rate of depression among adolescents was 40.7%, and the detection rate of anxiety was 24.1% [45], which may be related to the differences in the scales used and the regions, but all of these findings indicate that adolescent mental health needs to be emphasized by schools and families. This study also revealed that mental health problems were more prevalent among female adolescents than male adolescents. This may be related to biological differences between male adolescents and female adolescents, societal expectations and pressures on female adolescents, and female adolescents’ own sensitivity to external pressures [46,47]. Therefore, schools and families should pay more attention to female adolescents’ mental health and motivate them to develop in a positive direction.

### 4.2. Family Functioning and Adolescent Mental Health

The results of this study showed that family functioning has a direct association with adolescents’ mental health. This finding is consistent with the findings of Xu, Fang, Zhang, Lin, and Sun and Chen, Wang, and Yang that adolescents with poorer family functioning are more likely to have mental health problems such as depression and anxiety [48,49]. This study also further validated the family systems theory. The family has been found to be the initial and lifelong environment that ensures physical and mental health, as well as a core component of the social support system. Normal family functioning provides favorable environmental conditions for the physical, mental, and social health of family members [50]. The better the overall functioning of the family system (e.g., emotional responsiveness, communication), the better the psychological state and behavioral performance of the adolescent, which reduces the incidence of depression and other mental health problems [51]. Thus, good family functioning allows adolescents to feel the warmth of their parents, contributes to the development of positive emotions, and reduces the occurrence of mental health problems.

### 4.3. The Mediating Role of Bullying Victimization and Resilience

The study results revealed that adolescents who experience poor family functioning are more likely to become victims of bullying, which can lead to the development of psychological problems. This finding is consistent with the research conducted by Husky et al. [52]. Poorly functioning families may be unable to provide adequate social interactions and emotional support, which can result in adolescents lacking the ability to express themselves socially and emotionally. As a result, they may exhibit more passive behavior and greater vulnerability in interpersonal interactions, leading to maladjustment and a lack of trust in others. These factors may contribute to their increased likelihood of being bullied. Research suggests that adolescents who grow up in harmonious, warm, loving, and supportive family environments are more likely to develop positive interpersonal skills, empathy, self-control, and effective coping mechanisms. These skills can help prevent and reduce bullying in schools [53]. Unhealthy family environments, such as poor family functioning, family conflict, violence, or abuse, may have a negative impact on adolescents and increase their risk of being bullied.

Resilience also plays a crucial mediating role in family functioning and adolescent mental health. Family functioning has a significant impact on the development of an individual’s resilience, as well as teaching skills to cope with adversity and stress [54]. Positive communication, conflict resolution, and problem solving within the family can help individuals learn how to face challenges, adapt to change, and maintain emotional stability. These skills can be useful when individuals face stress and adversity, as they can improve their resilience and coping abilities [51]. Individuals with higher levels of resilience are better equipped to cope with psychological stress, setbacks, and trauma. They are also better able to regulate negative emotions and emotional distress, recover from difficult situations, and face challenges positively. This reduces the risk of mental health problems [55].

This study also revealed a mediating role between family functioning and adolescent mental health, where family functioning influences resilience and ultimately mental health by influencing the occurrence of bullying victimization. However, this relationship was only significant for male adolescents after the sample was divided by gender. This could be attributed to differences in the reporting of bullying behavior between male and female adolescents. Male and female adolescents experience different types of bullying, with male adolescents more likely to experience direct bullying and female adolescents more likely to experience indirect bullying [56]. Direct bullying victimization may have a greater impact on an individual’s resilience, which can subsequently affect their mental health. Therefore, it is important to pay attention to all forms of bullying in schools, including differences in bullying between male adolescents and female adolescents, to protect their mental health.

### 4.4. Gender Differences

Gender differences were found in the effects of family functioning on depression/anxiety, which is consistent with current views. Surprisingly, there were no gender differences in the mediating role of bullying victimization in the relationship between family functioning and depression/anxiety, which is consistent with a study of 1685 adolescents in Mexico [57]. Therefore, we hypothesize that bullying in schools poses the same threat to the mental health of male adolescents and female adolescents when adolescents have good family functioning (the mean scores for family functioning in this study were greater than 6, which is considered good family functioning). Future research may confirm this proposition.

Additionally, this study revealed gender disparities in the mediating impact of resilience on the correlation between family functioning and mental health. Specifically, family functioning is more likely to impact the resilience of male adolescents, leading to the emergence of mental health issues. Female adolescents may be more inclined to express their emotions and seek social support when facing stress and adversity [58]. However, this may not be the case for boys. Further research is necessary to gain insight into the specific causes of this phenomenon.

### 4.5. Limitations

Several limitations of the study should be mentioned. First, bullying victimization occurs at all stages of student life, but this study only included students from four high schools in central China, which limits its representativeness. The scope of the study subjects and regions can be expanded in future research. Second, the data are based on self-reports by adolescents, so there may be some reporting errors. Finally, this study used cross-sectional survey research, which cannot determine the causal relationships between variables. A longitudinally designed study is necessary for this purpose.

## 5. Conclusions

Although the findings need to be replicated and extended to longitudinal studies, this study provides preliminary evidence that bullying victimization and resilience not only independently mediate, but also chain mediate, the relationship between family functioning and adolescent mental health. Gender differences indicate that family functioning is more likely to influence resilience and, thus, mental health among male adolescents. In addition, upon dividing the sample into male and female groups, it was discovered that the chain-mediated effect between bullying victimization and resilience mediated the relationship between family functioning and mental health problems among male adolescents, but not among female adolescents. Therefore, efforts must be made at the family, school, and individual levels to improve the mental health of adolescents. It is particularly important to focus on the influence of family on adolescent bullying victimization and personal traits.

## Figures and Tables

**Table 1:** Comparison of Depression and Anxiety Scores with Various Demographic Variables.

Variables	n (%)	Depression	Anxiety
Gender			
Male	2347 (54.3)	4.41 ± 5.15	4.04 ± 4.65
Female	1972 (45.7)	5.64 ± 5.31	5.53 ± 4.72
t		−7.681	−10.399
*p*		<0.001	<0.001
Grade			
First-year senior	1987 (46.0)	5.24 ± 5.14	5.09 ± 4.93
Second-year senior	1663 (38.5)	4.64 ± 5.16	4.32 ± 4.55
Third-year senior	669 (15.5)	5.01 ± 5.00	4.59 ± 4.53
F		5.889	12.231
*p*		<0.01	<0.001
One-child family			
Yes	2732 (63.3)	4.89 ± 5.29	4.67 ± 4.80
No	1587 (36.7)	5.12 ± 5.20	4.80 ± 4.63
t		−1.392	−0.919
*p*		>0.05	>0.05
Family economy			
Good	1207 (27.9)	4.20 ± 4.77	4.29 ± 4.64
General	2804 (64.9)	5.02 ± 5.16	4.70 ± 4.58
Poor	308 (7.1)	7.61 ± 6.86	6.59 ± 5.95
F		37.499	20.232
*p*		<0.001	<0.001
Bullying victimization			
Yes	271 (6.3)	8.71 ± 6.57	7.85 ± 5.83
No	4048 (93.7)	4.72 ± 5.06	4.51 ± 4.58
t		−9.806	−9.241
*p*		<0.001	<0.001

**Table 2:** Correlations among the primary variables in the study.

	1	2	3	4	5
1. Family functioning					
2. Bullying victimization	−0.100 **				
3. Resilience	0.394 **	−0.106 **			
4. Depression	−0.431 **	0.184 **	−0.459 **		
5. Anxiety	−0.382 **	0.171 **	−0.439 **	0.794 **	

** *p* < 0.01.

**Table 3:** Direct and indirect effects in the model of family functioning and depression, mediated by bullying victimization and resilience.

Effects	Estimate	S.E.	95% CI		*p*-Value
			Lower	Upper	
**Direct effect**					
Family functioning → Depression	−0.305	0.017	−0.338	−0.272	<0.001
**Indirect effects**					
Family functioning → Bullying victimization → Depression	−0.013	0.003	−0.020	−0.009	<0.001
Family functioning → Resilience → Depression	−0.118	0.008	−0.134	−0.102	<0.001
Family functioning → Bullying victimization → Resilience → Depression	−0.002	0.001	−0.004	−0.001	<0.001

**Table 4:** Direct and indirect effects in the model of family functioning and anxiety, mediated by bullying victimization and resilience.

Effects	Estimate	S.E.	95% CI		*p*-Value
			Lower	Upper	
**Direct effect**					
Family functioning → Anxiety	−0.261	0.018	−0.297	−0.225	<0.001
**Indirect effects**					
Family functioning → Bullying victimization → Anxiety	−0.013	0.003	−0.019	−0.008	<0.001
Family functioning → Resilience → Anxiety	−0.116	0.008	−0.133	−0.101	<0.001
Family functioning → Bullying victimization → Resilience → Anxiety	−0.002	0.001	−0.004	−0.001	<0.001

**Table 5:** Comparison of depression mediation models by gender.

Effects	Male			Female		
	Estimate	SE	*p*-Value	Estimate	SE	*p*-Value
**Direct effect**						
Family functioning → Depression	−0.328	0.022	<0.001	−0.268	0.025	<0.001
**Indirect effects**						
Family functioning → Bullying victimization → Depression	−0.017	0.004	<0.001	−0.010	0.004	<0.01
Family functioning → Resilience → Depression	−0.094	0.010	<0.001	−0.156	0.013	<0.001
Family functioning → Bullying victimization → Resilience → Depression	−0.003	0.001	<0.001	−0.002	0.001	>0.05

**Table 6:** Comparison of anxiety mediation models by gender.

Effects	Male			Female		
	Estimate	SE	*p*-Value	Estimate	SE	*p*-Value
**Direct effect**						
Family functioning → Anxiety	−0.304	0.024	<0.001	−0.205	0.026	<0.001
**Indirect effects**						
Family functioning → Bullying victimization → Anxiety	−0.016	0.004	<0.001	−0.010	0.004	<0.01
Family functioning → Resilience → Anxiety	−0.089	0.010	<0.001	−0.162	0.014	<0.001
Family functioning → Bullying victimization → Resilience → Anxiety	−0.003	0.001	<0.001	−0.002	0.001	>0.05

## Data Availability

The data presented in this study are available on request from the corresponding author.

## References

[1] Bor W., Dean A.J., Najman J., Hayatbakhsh R. (2014). Are child and adolescent mental health problems increasing in the 21st century? A systematic review. Aust. N. Z. J. Psychiatry.

[2] Collier C. (2012). Adolescent mental health: Interna. CMAJ.

[3] Chen J., Liao W. (2005). Childhood non-contact corporal punishment revealed in the questionnaire survey of technical secondary school students. Chin. Ment. Health J..

[4] Ma L., Mazidi M., Li K., Li Y., Chen S., Kirwan R., Zhou H., Yan N., Rahman A., Wang W. (2021). Prevalence of mental health problems among children and adolescents during the COVID-19 pandemic: A systematic review and meta-analysis. J. Affect. Disord..

[5] Bisson K.H. (2017). The Effect of Anxiety and Depression on College Students’ Academic Performance: Exploring Social Support as a Moderator. Master’s Thesis.

[6] Bauer M.E., Teixeira A.L. (2021). Neuroinflammation in mood disorders: Role of regulatory immune cells. Neuroimmunomodulation.

[7] Bronfenbrenner U., Ceci S.J. (1994). Nature-nuture reconceptualized in developmental perspective: A bioecological model. Psychol. Rev..

[8] Steinberg L., Morris A.S. (2001). Adolescent development. Annu. Rev. Psychol..

[9] Cook W.L., Kenny D.A. (2006). Examining the validity of self-report assessments of family functioning: A question of the level of analysis. J. Fam. Psychol..

[10] Qiankun H., Shengli T. (2022). Effects of family function and stress feeling on the mental health of adolescents. Chin. J. Child Health Care.

[11] Tamplin A., Goodyer I.M., Herbert J. (1998). Family functioning and parent general health in families of adolescents with major depressive disorder. J. Affect. Disord..

[12] Oltean I.I., Perlman C., Meyer S., Ferro M.A. (2020). Child mental illness and mental health service use: Role of family functioning (family functioning and child mental health). J. Child Fam. Stud..

[13] Tamplin A., Gooyer I. (2001). Family functioning in adolescents at high and low risk for major depressive disorder. Eur. Child Adolesc. Psychiatry.

[14] Bögels S.M., Brechman-Toussaint M.L. (2006). Family issues in child anxiety: Attachment, family functioning, parental rearing and beliefs. Clin. Psychol. Rev..

[15] Olweus D. (1993). Bully/victim problems among schoolchildren: Long-term consequences and an effective intervention program. Mental Disorder and Crime.

[16] Stevens V., De Bourdeaudhuij I., Van Oost P. (2002). Relationship of the family environment to children’s involvement in bully/victim problems at school. J. Youth Adolesc..

[17] Zych I., Farrington D.P., Ttofi M.M. (2019). Protective factors against bullying and cyberbullying: A systematic review of meta-analyses. Aggress. Violent Behav..

[18] Eşkisu M. (2014). The relationship between bullying, family functions, perceived social support among high school students. Procedia-Soc. Behav. Sci..

[19] Chen J.-K., Wang S.-C., Chen Y.-W., Huang T.-H. (2021). Family climate, social relationships with peers and teachers at school, and school bullying victimization among third grade students in elementary schools in Taiwan. Sch. Ment. Health.

[20] Källmén H., Hallgren M. (2021). Bullying at school and mental health problems among adolescents: A repeated cross-sectional study. Child Adolesc. Psychiatry Ment. Health.

[21] Masood N., Okazaki S., Takeuchi D.T. (2009). Gender, family, and community correlates of mental health in South Asian Americans. Cult. Divers. Ethn. Minor. Psychol..

[22] Kim Y.K., Kim Y.J., Maleku A., Moon S.S. (2019). Typologies of peer victimization, depression, and alcohol use among high school youth in the United States: Measuring gender differences. Soc. Work Public Health.

[23] Bolier L., Haverman M., Westerhof G.J., Riper H., Smit F., Bohlmeijer E. (2013). Positive psychology interventions: A meta-analysis of randomized controlled studies. BMC Public Health.

[24] Dou D., Shek D.T., Tan L., Zhao L. (2023). Family functioning and resilience in children in mainland China: Life satisfaction as a mediator. Front. Psychol..

[25] Davydov D.M., Stewart R., Ritchie K., Chaudieu I. (2010). Resilience and mental health. Clin. Psychol. Rev..

[26] Nam B., Kim J.Y., DeVylder J.E., Song A. (2016). Family functioning, resilience, and depression among North Korean refugees. Psychiatry Res..

[27] Anderson J.R., Mayes T.L., Fuller A., Hughes J.L., Minhajuddin A., Trivedi M.H. (2022). Experiencing bullying’s impact on adolescent depression and anxiety: Mediating role of adolescent resilience. J. Affect. Disord..

[28] Weitkamp K., Seiffge-Krenke I. (2019). The association between parental rearing dimensions and adolescent psychopathology: A cross-cultural study. J. Youth Adolesc..

[29] Lin L.-Y., Chien Y.-N., Chen Y.-H., Wu C.-Y., Chiou H.-Y. (2022). Bullying experiences, depression, and the moderating role of resilience among adolescents. Front. Public Health.

[30] Chang F.C., Lee C.M., Chiu C.H., Hsi W.Y., Huang T.F., Pan Y.C. (2013). Relationships among cyberbullying, school bullying, and mental health in Taiwanese adolescents. J. Sch. Health.

[31] Katz-Wise S.L., Sarda V., Line E.C., Marchwinski B., Budge S.L., Godwin E.G., Moore L., Ehrensaft D., Rosal M.C., Thomson K.A. (2024). Longitudinal Family Functioning and Mental Health in Transgender and Nonbinary Youth and Their Families. J. Child Fam. Stud..

[32] Xu M., Wu Q., Zhu H. Research on the emotional balance, resilience and mental health of adolescents during the epidemic. Proceedings of the 2nd International Conference on Computer Vision, Image, and Deep Learning.

[33] Smilkstein G. (1978). The family APGAR: A proposal for a family function test and its use by physicians. J. Fam. Pract..

[34] Lv F., Gu Y. (1995). Family APGAR questionnaire and its clinical application. Handb. Hosp. Manag..

[35] Zhang W., Wu J., Jones K. (1999). Revision of the Chinese version of olweus child bullying questionnaire. Psychol. Dev. Educ..

[36] Campbell-Sills L., Stein M.B. (2007). Psychometric analysis and refinement of the connor–davidson resilience scale (CD-RISC): Validation of a 10-item measure of resilience. J. Trauma. Stress Off. Publ. Int. Soc. Trauma. Stress Stud..

[37] Cheng C., Dong D., He J., Zhong X., Yao S. (2020). Psychometric properties of the 10-item Connor–Davidson Resilience Scale (CD-RISC-10) in Chinese undergraduates and depressive patients. J. Affect. Disord..

[38] Kroenke K., Spitzer R.L., Williams J.B. (2001). The PHQ-9: Validity of a brief depression severity measure. J. Gen. Intern. Med..

[39] Min B.-q., Zhou A.-h., Liang F., Jia J. (2013). Clinical application of Patient Health Questionnaire for self-administered measurement (PHQ-9) as screening tool for depression. J. Neurosci. Ment. Health.

[40] Spitzer R.L., Kroenke K., Williams J.B., Löwe B. (2006). A brief measure for assessing generalized anxiety disorder: The GAD-7. Arch. Intern. Med..

[41] Moss S. (2009). Fit indices for structural equation modeling. Front. Psychol..

[42] Marsh H.W., Hau K.-T., Wen Z. (2004). In search of golden rules: Comment on hypothesis-testing approaches to setting cutoff values for fit indexes and dangers in overgeneralizing Hu and Bentler’s (1999) findings. Struct. Equ. Model..

[43] Fuller C.M., Simmering M.J., Atinc G., Atinc Y., Babin B.J. (2016). Common methods variance detection in business research. J. Bus. Res..

[44] Huang X.-X., Li Y.-T., Chen J.-H., Ma J.-J., Cong E.-Z., Xu Y.-F. (2023). The influence of family structure on depression and anxiety symptoms in adolescents: The mediating role of emotional neglect. Zhongguo Dang Dai Er Ke Za Zhi Chin. J. Contemp. Pediatr..

[45] Liu S., Chen X., Li Y., Yuan X., Yu H., Fang M. (2021). Investigation of depression and anxiety and their influencing factors among adolescents in Beijing during the coronavirus disease 2019 epidemic. J. Cap. Med. Univ..

[46] Buli B., Larm P., Nilsson K., Giannotta F. (2021). Trends in adolescent mental health problems: Differences by SES and gender. Eur. J. Public Health.

[47] Knowles G., Gayer-Anderson C., Beards S., Blakey R., Davis S., Lowis K., Stanyon D., Ofori A., Turner A., Pinfold V. (2021). Mental distress among young people in inner cities: The Resilience, Ethnicity and AdolesCent Mental Health (REACH) study. J. Epidemiol. Community Health.

[48] Xu J., Fang X., Zhang J., Lin D., Sun L. (2008). Effect mechanism of family functioning on adolescent’s emotional problem. Psychol. Dev. Educ..

[49] Chen B., Wang W., Yang S. (2023). The impact of family functioning on depression in college students: A moderated mediation model. J. Affect. Disord..

[50] Jing H., Hui-chang C., Xin-yin C. (2009). The prediction of Behavioral Inhibition of the Children from Two to Seven Years Old for Problem Behavior and School Adjustment. Psychol. Dev. Educ..

[51] FletcherD S. (2013). Psychologicalresilience: Areviewand critiqueofdefinitions, concepts, andtheory. Eur. Psychol..

[52] Husky M.M., Delbasty E., Bitfoi A., Carta M.G., Goelitz D., Koç C., Lesinskiene S., Mihova Z., Otten R., Kovess-Masfety V. (2020). Bullying involvement and self-reported mental health in elementary school children across Europe. Child Abus. Negl..

[53] Arseneault L., Bowes L., Shakoor S. (2010). Bullying victimization in youths and mental health problems:‘Much ado about nothing’?. Psychol. Med..

[54] Fletcher D., Sarkar M. (2012). A grounded theory of psychological resilience in Olympic champions. Psychol. Sport Exerc..

[55] Walsh F. (2003). Family resilience: A framework for clinical practice. Fam. Process.

[56] Silva-Rocha N., Soares S., Brochado S., Fraga S. (2020). Bullying involvement, family background, school life, and well-being feelings among adolescents. J. Public Health.

[57] Nuñez-Fadda S.M., Castro-Castañeda R., Vargas-Jiménez E., Musitu-Ochoa G., Callejas-Jerónimo J.E. (2022). Impact of bullying—Victimization and gender over psychological distress, suicidal ideation, and family functioning of mexican adolescents. Children.

[58] Bibi A., Lin M., Brailovskaia J., Margraf J. (2023). Mental health of university students of Pakistan and Germany and the right to health care. Int. J. Hum. Rights Healthc..

